# Accelerated vs. conventionally fractionated adjuvant radiotherapy in high-risk head and neck cancer: a meta-analysis

**DOI:** 10.1186/s13014-018-1133-8

**Published:** 2018-10-04

**Authors:** Christiane Matuschek, Jan Haussmann, Edwin Bölke, Stephan Gripp, Patrick J. Schuler, Bálint Tamaskovics, Peter Arne Gerber, Freddy-Joel Djiepmo-Njanang, Kai Kammers, Christian Plettenberg, Bahar Anooshahr, Klaus Orth, Wilfried Budach

**Affiliations:** 1 Department of Radiotherapy and Radiooncology, Medical Faculty, Heinrich Heine University, Moorenstr. 5, 40225 Dusseldorf, Germany; 2 Department of Oto-Rhino-Laryngology, Head and Neck Surgery, Ulm University Medical Center, Ulm, Germany; 3 Department of Dermatology, Medical Faculty Heinrich Heine University Dusseldorf, Dusseldorf, Germany; 4 Division of Biostatistics and Bioinformatics, Department of Oncology, The Sidney Kimmel Comprehensive Cancer Center at Johns Hopkins, The Johns Hopkins University School of Medicine, Baltimore, MD USA; 5 Department of Oto-Rhino-Laryngology, Head and Neck Surgery, Heinrich Heine University Dusseldorf, Dusseldorf, Germany; 6 Vincere Cancer Center, Scottsdale, AZ USA

**Keywords:** Radiation therapy, Head and neck cancer, High risk, Adjuvant therapy, Conventional fractionation, Accelerated fractionation

## Abstract

**Background:**

Adjuvant radiotherapy in advanced head and neck squamous cell cancer (HNSCC) reduces the risk of local-regional failure and most likely increases the survival rate. Patients at high risk for tumor recurrence may benefit from more aggressive altered fractionation schedules in order to reduce the overall time from surgery to completion of radiotherapy. Here, we reviewed the results of six randomized trials addressing the above hypothesis.

**Methods:**

In the six trials of interest, a total of 988 patients with locally advanced HNSCC were randomly assigned to receive either accelerated or conventionally fractionated adjuvant radiotherapy. Hazard ratios (HR) were extracted from available publications for local-regional control, distant metastasis as well as overall-, cancer specific- and disease-free survival. Meta-analysis of the effect sizes was performed using fixed and random effect models. Acute and late side effects were categorized and summarized for comparison.

**Results:**

Accelerated radiotherapy did not improve the loco-regional control (*n* = 988, HR = 0.740, CI = 0.48–1.13, *p* = 0.162), progression-free survival (HR = 0.89, CI = 0.76–1.04, *p* = 0.132) or overall survival (HR = 0.88, CI = 0.75–1.04, *p* = 0.148) significantly. Acute confluent mucositis occurred with significant higher frequency with accelerated radiotherapy. Late side effects did not differ significantly in either group.

**Conclusion:**

Accelerated radiotherapy does not result in a significant improvement of loco-regional control or overall survival in high-risk patients. Acute but not late radiation toxicity were more frequent with the accelerated RT technique. In clinical practice accelerated postoperative radiation therapy might be a suitable option only for a subset of patients.

## Background

Head and neck squamous cell carcinoma (HNSCC) is a common tumor and accounts for approximately 3% of all cancers in the United States. About 40% of patients have locally advanced disease at the time of diagnosis. Surgery, radiation therapy (RT), or both have been used for decades to improve loco-regional control (LRC) and overall survival (OS). The most common schedule for normofractionated RT alone is given as 1.6–2.0 Gy per fraction per day, 5 days a week, for 6–7 weeks. Despite all efforts, the prognosis of patients with locally advanced HPV-negative HNSCC is still disappointing, with 5-year OS rates of 30–35% [1–11].

The role of unconventionally fractionated RT in HNSCC has been studied in numerous trials within different schedules which are usually separated into hyperfractionated (HF) where the treatment is administered in two smaller fractions twice per day and accelerated fractionation (AF) where the overall treatment time is reduced by applying more than 5 fractions per week [12–14].

The rational for this approach is that a shortening of overall treatment time might result in improved local tumor control rates as it counteracts tumor repopulation. Secondly smaller fractions are also believed to result in a reduction in late toxicities as normal tissue, in contrast to tumors, are able to repair RT-induced damages within 6 h [1–4, 15–17]. Thirdly there are also economic and patient convenience arguments to shorten the overall treatment time on a linear accelerator time slot. As surgical interventions have significant impact on morbidity and quality of life it is important to study the influence of different fractionation regimes on adverse events in the postoperative setting [5].

The MARCH meta-analysis published by Bourhis et al. demonstrated that altered fraction schedules are a valuable alternative to chemoradiation as they provide similar gains in reduction of overall mortality compared to standard radiation alone in the definitive therapeutic setting. However between the subgroups of altered fractionation only the hyperfractionated, and not the accelerated, schedules appear to provide a substantial mortality benefit beyond a reduction in local recurrence [14].

However it is currently not established whether this is also true for the postoperative / adjuvant situation. This setting is accompanied by a smaller tumor volume and density, different tumor microenvironment, local inflammation and a potentially different radiobiology i.e. lesser extent of hypoxic cells. These observations allow an investigation of accelerated schedules in the postoperative setting and to the best of our knowledge no meta-analysis has focused on this aspect yet. The results of six randomized trials addressing this question in adjuvant RT were retrieved and analyzed [6–11, 18].

## Methods

We thoroughly investigated the electronic databases MEDLINE and EMBASE from inception through July 23, 2017, with no restriction for language or publication status. We also searched the Evidence Based Medicine Reviews database combining searches of Cochrane Database of Systematic Reviews, Database of Abstracts of Reviews of Effects, Cochrane CENTRAL, Cochrane Methodology Register, Health Technology Assessment, NHS Economic Evaluation Database, and ACP Journal Club. Patients with locally advanced HNSCC were randomly assigned to receive either accelerated or conventionally fractionated postoperative RT. Studies that included concurrent or sequential chemotherapy were not allowed. Published hazard ratios and hazard ratios extracted from available survival curves were the basis of the meta-analysis. Additionally, we extracted and categorized the published acute and late side effects from the published papers [6–11, 18, 19]. The idea for this meta-analysis was originally generated in 2016. By the time the literature search was completed and the manuscript was prepared the individual patient meta-analysis by Lacas et al. was published and generated superior estimates of the investigated effect size on overall survival for the include trials [18]. Moreover the extraction of these published data allowed the inclusion of multiple other endpoints beyond locoregional control and overall survival. Additionally the CHARTWEL study, as an unpublished trial, could be included which was otherwise unavailable.

Heterogeneity between trials was assessed using Chi-square test and I statistics. Estimations of the pooled effect sizes were performed using a fixed effect model. When I-statistics showed significant heterogeneity (p = < 0.1) a random effect model was applied. Meta-analyses of the effect sizes on locoregional-, local- and regional recurrence, distant failure, progression-free survival, overall survival, cancer- and non-cancer mortality were performed using fixed or random effects models based on hazard ratios and their standard errors using the Microsoft Excel add-in Meta-XL (Version 5.3).

As hazard ratios of clinical endpoints were extracted from the analysis of Lacas et al. all considerations in their paper apply equally to our results [18]. The most important ones are: overall survival was defined as time from randomization to death from any cause. Living patients were censored at the time of the last follow-up. Cancer mortality is defined as death with previous diagnosis of progression and of the treated head and neck cancer as well as death from unknown cause within 5 years after randomization. Time from randomization to first local or distant recurrence or death from any cause was defined as progression-free survival. All events were recorded as first events.

The Cairo 1990 trial reported only combined locoregional recurrence and was not included in the separate analysis of local and regional recurrence [11]. The analysis of locoregional failure consisted of pooled effect sizes of both endpoints from the other trials by a fixed effect model as no heterogeneity was observed. Statistical difference level was set at *p*-values lower than 0.05 for all evaluations.

## Results

We found five publications testing the effect of accelerated vs conventional fractionated postoperative RT in high-risk HNSCC patients [6–11]. Extraction of the single trial data from Lacas et al. allowed the inclusion of six trials [18]. All staging was defined by pathology.

The first paper, published in 2005, was a multicenter Phase III study by Sanguineti et al. [8]. Between 1994 and 2000, 226 patients were enrolled. They found a 2-year LRC of 80% +/− 4% for conventional RT and 78% +/− 5% for accelerated RT (*p* = 0.52). The 2-year OS were 67% +/− 5% for conventional and 64% +/− 5% for accelerated RT (*p* = 0.84). No difference in the clinical outcome among the two treatment arms was detected in a multivariate analysis. Nevertheless, interaction analysis with median values as cut-offs showed a tendency for improved LRC in patients treated with accelerated fractionation (HR = 0.5, CI = 0.2–1.1). Fifty percent of patients treated with accelerated RT had confluent mucositis, compared with only 27% of those treated with conventional fractionation (*p* = 0.006). However, the duration of mucositis did not differ in the two groups. Actuarial Grade 3+ late toxicity rates at 2 years were 18% +/− 4% and 27% +/− 6% for conventional and accelerated RT (*p* = 0.10) [8].

The second paper was published in 2001 by K. Ang et al. from MD Anderson [9]. The group performed a multi-institutional, prospective, randomized trial. In this trial they investigated multiple variables including pathologic risk features, the RT-dose required in the adjuvant setting and the the impact of accelerating RT using a concomitant boost schedule as well as the importance of the overall combined treatment duration on the treatment outcome. Two hundred thirteen patients with advanced disease were divided into three groups based on a set of pathologic risk features. The high-risk group received 63 Gy, by random assignment, for 5 weeks (*n* = 76) or 7 weeks (*n* = 75). Patients were irradiated with standard protocols applicable to the disease site and expected areas of tumor spread. The endpoints were LRC, OS and morbidity. Patients with high-risk patients tended to have a better LRC and OS when postoperative RT was administered in 5 rather than 7 weeks. A longer interval between surgery and postoperative RT in the 7-week timetable led to considerably lower LRC (*p* = 0.03) and OS (*p* = 0.01) rates. As a result, the cumulative length of combined therapy significantly impacted the LRC (*p* = 0.005) and OS (p = 0.03) rates. The shorter radiation time, achieved by administering the boost simultaneously did not increase toxicities [9].

Awwad et al. published the third paper in the *British Journal of Cancer* [11]. The trial included 70 patients who had had radical surgery for (T2/N1-N2) or (T3–4/any N) squamous cell carcinoma of the oral cavity, larynx, and hypopharynx. Patients were assigned at random to receive either (a) accelerated hyperfractionation: 46.2 Gy per 12 days, 1.4 Gy per fraction, three fractions per day with 6 h interfraction interval, treating 6 days per week or (b) conventional fractionation: 60 Gy per 6 weeks, 2 Gy per fraction, treating 5 days per week. The researchers found a significant improvement in 3-year LRC in the accelerated hyperfractionation as opposed to conventional fractionation (88 +/− 4% vs 57+/− 9%) (*p* = 0.01). There was, however, no significant difference in the OS (60 +/− 10% vs 46 +/− 9%) (*p* = 0.29). Further, the accelerated hyperfractionated radiotherapy was most beneficial when it started within 6 weeks after surgery and the total treatment time was completed within 10 weeks. Mucositis emerged earlier and was more severe in the accelerated hyperfractionation group. Accelerated hyperfractionation caused more xerostomia, fibrosis and edema. In comparison to conventional fractionation, accelerated hyperfractionation did not seem to offer a survival advantage in patients with rapidly growing tumors. A better local control, however, was achieved in these patients. For slow growing tumors, there was no significant difference in tumor control and survival rate between the two radiation schedules [11].

Suwinski performed a randomized clinical trial on 7-days-a-week postoperative RT for high-risk HNSCC [10]. Between 2001 and 2004, 279 patients with high-risk squamous cell cancer of the larynx (*n* = 158) or cancer of the oral cavity/oropharynx (*n* = 121) were registered. Patients received 63 Gy in 35 fractions given 5-days-a-week (*n* = 140: conventional fraction group) or 7-days-a-week (*n* = 139: accelerated group). Acute and late toxicity were considered acceptable, although the proportion of patients with confluent mucositis was higher in the accelerated group compared to conventional fractionation (60% vs. 33.3%). The actuarial 3-year LRC were not significantly different with 64% for conventional vs. 70% for accelerated fractionation (*p* = 0.32) for the whole population. However accelerated treatment yielded a statistically significant improvement in the 3-year LRC for patients with cancer of the oropharynx/oral cavity (74% vs. 53% conventional fractionated, *p* = 0.02). No improvement was found for the local-regional control for patients with cancer of the larynx (*p* = 0.46) [10].

At the European Society for Radiotherapy and Oncology annual meeting in 2015, Langendijk et al. presented their final trial results [6, 7]. Patients with high-risk advanced HNSCC (i.e., positive surgical margins and/or extranodal spread) treated with curative surgery were randomly assigned to receive either standard postoperative RT (2 Gy/fraction/day, 5 days/week to 66 Gy/33 fractions/7 weeks) or postoperative accelerated RT (2 Gy/fraction/day, 5 days per week, to 20 Gy followed by 1.8 Gy/fraction/day and 1.3 Gy/fraction per day to a boost field as a second daily treatment to 66.5 Gy/40 fractions/5 weeks). Endpoints were LRC, OS, acute and late toxicity, and quality of life. A total of 148 patients were registered in this trial (74 pts. for conventional radiotherapy and 74 pts. for the accelerated group). No significant changes were found for acute and late toxicity, although there was a trend towards increased need for pain medication among patients in the accelerated group. After 3 years, the LRC rate was 77% in the accelerated group compared to 76% for patients with conventional fractionation (HR = 0.86, CI = 0.42–1.76; *p* = 0.68). The 3-year OS was not significantly different for the accelerated group vs. the conventionally treated group (71% vs. 63% HR = 0.93, CI = 0.54–1.61; *p* = 0.81) [7].

The data from the unpublished CHARTWEL trial was available from the updated MARCH meta-analysis [18]. Between 2001 and 2005 114 eligible patients with stage I-IV HNSCC of the oral cavity, oropharynx, hypopharynx, larynx or other sites were randomized to CF RT of 60–64 Gy in 6–6.5 weeks or 51–54 Gy with 1.5 Gy three times daily for 2.4 weeks. Trials results are reported after a median follow-up of 4.8 years.

**Fig. 1:**
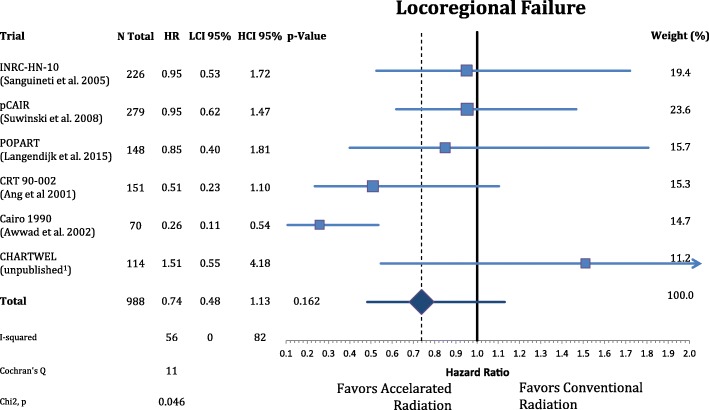
Forest plot of comparison of locoregional failure between accelerated and conventional radiation using random effect model. Horizontal bars indicate the amount of variation (95% confidence intervals of the parameter estimates). Sizes of squares indicate weight in the pooled effect size

**Fig. 2:**
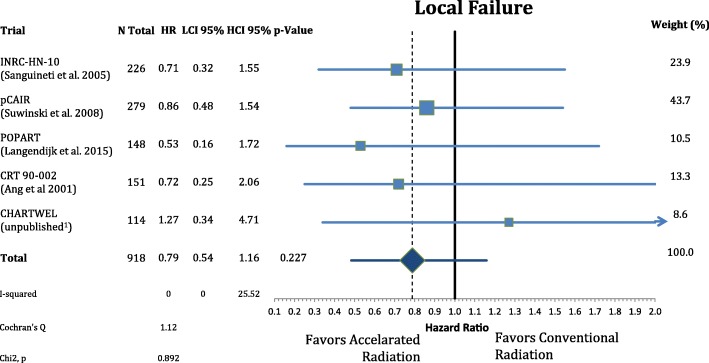
Forest plot of comparison of local failure between accelerated and conventional radiation using fixed effect model

**Fig. 3:**
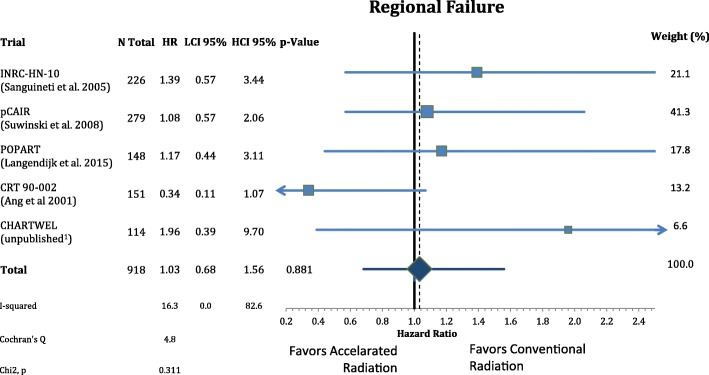
Forest plot of comparison of regional failure between accelerated and conventional radiation using fixed effect model

**Fig. 4:**
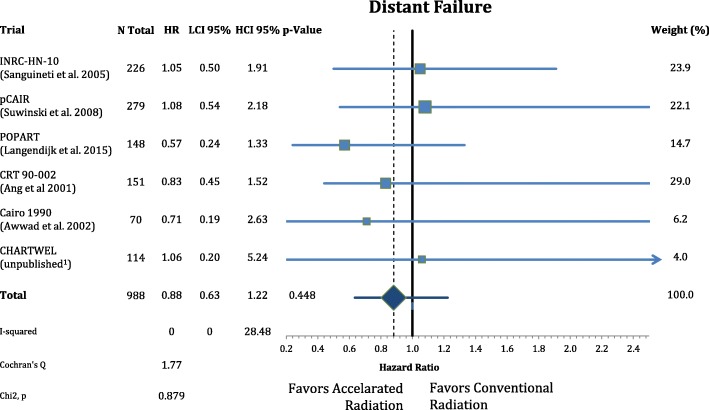
Forest plot of comparison of distant failure between accelerated and conventional radiation using fixed effect model

**Fig. 5:**
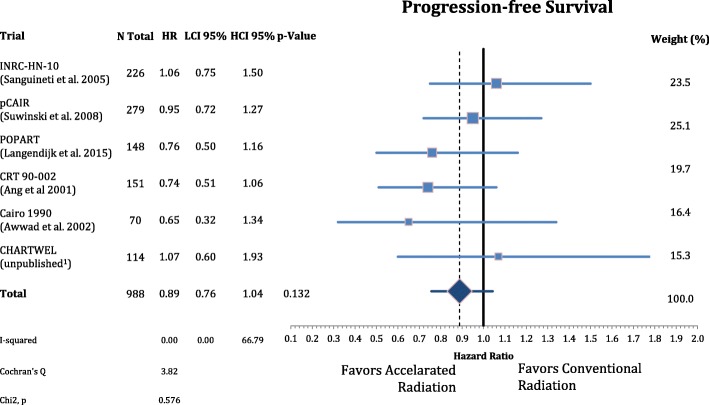
Forest plot of comparison of progression-free survival between accelerated and conventional radiation using fixed effect model

The patients’ characteristics and inclusion criteria are summarized in Tables 1 and 2. All trials included patients with high risk squamous cell carcinoma of the head and neck region randomized to AF or CF without concomitant chemotherapy in both arms. Median follow-up ranges from 3.8–13.8 years. Overall 988 patients were analyzed in eight different oncological endpoints. Figure 1 shows that postoperative accelerated RT did not result in a significant improvement of LRC (*n* = 988, HR: 0.740, CI = 0.48–1.13, *p* = 0.162). Differential analysis of local and regional control separately **(**Figs. 2 and 3**)** demonstrates a non-significant reduction in local recurrence (*n* = 918, HR = 0.79, CI = 0.54–1.16, *p* = 0.227) and no effect on regional control (n = 918, HR = 1.03, CI = 0.68–1.56, *p* = 0.881). Likewise there is also no difference in distant metastasis (n = 988, HR = 0.88, CI = 0.63–1.22, *p* = 0.448) in Fig. 4 and progression free survival (n = 988, HR = 0.89, CI = 0.76–1.04, *p* = 0.132) (Fig. 5**).**

**Table 1:** (Reported) Study Inclusion criteria and radiation schedules in the included trials. All Staging is defined by pathology

	Ang et al. 2001 [9]	Awwad et al. 2002 [11]	Langendijk et al. 2015 [7]	Sanguineti et al. 2005 [8]	Suwinski et al. 2008 [10]	CHARTWEL unpublished^a^
N patients	151	70	148	226	279	114
Median Follow-up (years; IQR)	13.8 (8.0–16.9)	3.8 (1.6–4.7)	6.3 (5.3–8.0)	4.5 (3.4–6.2)	7.2 (6.3–8-0)	4.8 (3.9–5.4)
Histology	squamous cell carcinoma					n.a.
Localisation	oral cavity / oropharynx / hypopharynx / larynx	oral cavity / hypopharynx / larynx	n.a.	oral cavity / oropharynx / hypopharynx / larynx	oral cavity / oropharynx / larynx	oral cavity / oropharynx / hypopharynx / larynx /other
ECOG PS	≤ 2	≤ 2	n.a.	≤ 2	≤ 1	≤ 2
Age (years)	n.a.	≤ 65	n.a.	18–80	n.a.	n.a.
Resection	planned	surgical resection without macroscopic residual (R0/R1)				n.a.
Definition of “High Risk” situation	ECE or any two of following: - > 1 nodal group - ≥2 positive nodes - > 3-cm node - Oral cavity - Microscopic pos. Margin - Perineural invasion (Pn1)	- T2/N1–2 or - T3–4/any N	- positive surgical margins and/or - extranodal spread (ECE)	one or more of following risk factors: - pT4 - close margin (< 3 mm) - V1 - L1 - pN2–3 - ECE - time from surgery: ≤ 8 w	all non-laryngeal tumors (except T1 N0) or R1 or ECE+ or pN2/3 or two of following minor risk factors: - close margin - V1 - G3 - Pn1	n.a.
Metastases	no distant metastases (M0)					n.a.
Other criteria	n.a.	liver, kidney and other function normal	n.a.	n.a.	n.a.	n.a.
Dose in CF Arm	1.8Gyx35Fx =63Gy 5×/week in 7 weeks	2Gyx30Fx = 60Gy 5×/week in 6 weeks	2Gyx33Fx = 66Gy 5×/week in 7 weeks	2Gyx30Fx = 60Gy 5×/week in 6 weeks	1.8Gyx35Fx = 63Gy 5×/week in 7 weeks	2Gyx30-32Fx = 60-64Gy 5×/week in 7 weeks
Dose in AF Arm	1.8Gyx15Fx = 27Gy;5×/week+ 2 × 1.8Gy/d × 10; 5×/week = 63Gy in 35Fx in 5 weeks	3 × 1,4Gy/d = 46,2Gy; 6×/week in 12 days	2Gy×10Fx = 20Gy;5×/week + (1,8Gy + 1,3Gy/d)×15 = 66,5Gy in 40 Fx; in 5 weeks	(1.8Gy + 1.4Gy/d)x5Fx +2Gyx15Fx + (2Gy + 1.6Gy/d)x5Fx = 64Gy in 35 Fx 5×/week in 5 weeks	1.8Gyx35Fx = 63Gy; 7×/week in 5 weeks	(1.5Gyx3/d)x30Fx = 51-54Gy; 5×/week in 2.4 weeks

*ECE* Extracapsular extension, *CF* Conventional fractionation, *AF* Accelerated fractionation, *n.a.* Not available, *Fx* Fractions

^a^Data from the CHARTWEL trial were retrieved from [18]

**Table 2:** Patient characteristics of the included trials

	Ang et al. 2001 [9]		Awwad et al. 2002 [11]		Langendijk et al. 2015 [7]		Sanguineti et al. 2005 [8]		Suwinski et al. 2008 [10]		CHARTWEL unpublished^a^
	*n* = 151		*n* = 70		*n* = 148		*n* = 226		*n* = 279		*n* = 114
	CF *n* = 75	AF *n* = 76	CF	AF	CF *n* = 74	AF *n* = 74	CF	AF	CF	AF	
Age (years)											
median (range)	57 (20–83)		50 (25–65)	50 (29–65)	n.a.		61.5 (30–82)		57 (36–77)		n.a.
Gender											
male	163		32	24	111		209		127	122	88
female	50		7	7	37		17		13	17	26
Performance status	(ECOG)										
0	23		0	0	n.a.	n.a.	28	32	279		74
1	114		23	13	n.a.	n.a.	80	73			37
2	14		16	18	n.a.	n.a.	5	8	0		3
3	0		0	0	n.a.	n.a.	0	0	0		
Tumor site											
oral cavity	80	14	14	14	n.a.	n.a.	25	19	59	62	n.a.
oropharynx	66	0	0	0	23		13	27			30
hypopharynx	29	5	5	7	n.a.	n.a.	20	31	0	0	n.a.
larynx	38	20	20	10	10		55	36	81	77	16.
TNM: tumor stage											
T1/T2	84		5	3	21		3	4	36	35	20
T3	129		22	18	n.a.	n.a.	43	56	104	104	n.a
T4			12	10	n.a.	n.a.	67	53			n.a.
TNM: nodal stage											
N0	90		25	16	n.a.	n.a.	41	39	54	39	n.a.
N1			14	15	n.a.	n.a.			86	100	
N2/N3	123						72	74			
Histological grade											
G1	n.a.	15	15	13	n.a.	n.a.	n.a.	n.a.	30	16	n.a.
G2	n.a.	20	20	16	n.a.	n.a.	n.a.	n.a.	66	79	n.a.
G3	n.a.	4	4	2	n.a.	n.a.	n.a.	n.a.	31	37	n.a.
uncertain	213	0	0	0	n.a.	n.a.	113	113	13	7	n.a.
Stage (UICC 7th ed.)											
I	0		2		21		1	1	n.a.	n.a.	20
II	9						2	3	n.a.	n.a.	
III	81		42		23		8	13	n.a.	n.a.	13
IV	103		26		104		102	96	n.a.	n.a.	62
unknown	20	39	39	31	n.a.	n.a.	0	0	140	139	n.a.
Positive (or close)	resection margins										
yes	n.a.	17	17	15	n.a.	n.a.	48	48	78	77	n.a.
no	n.a.	20	20	16	n.a.	n.a.	65	65	50	54	n.a.
uncertain	213	2	2	0	n.a.	n.a.	0	0	12	8	n.a.
Extracapsular extension											
yes	104	n.a.	n.a.	n.a.	n.a.	n.a.	38	37	17	27	n.a.
no	109	n.a.	n.a.	n.a.	n.a.	n.a.	75	76	83	67	n.a.
uncertain	0	39	39	31	n.a.	n.a.	0	0	40	45	n.a.

*CF* Conventional fractionation group, *AF* Accelerated fractionation group

^a^Data from the CHARTWEL trial were retrieved from [18]

**Fig. 6:**
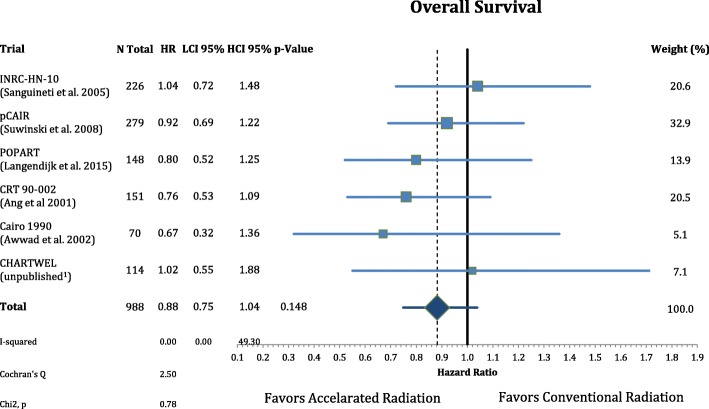
Forest plot of comparison of overall survival between accelerated and conventional radiation using fixed effect model

**Fig. 7:**
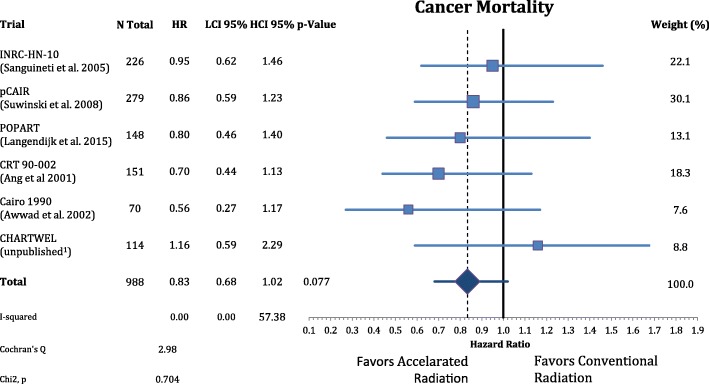
Forest plot of comparison of cancer mortality between accelerated and conventional radiation using fixed effect model

**Fig. 8:**
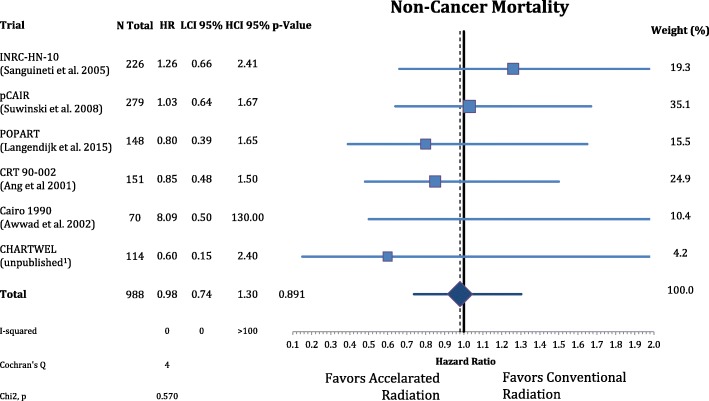
Forest plot of comparison of non-cancer mortality between accelerated and conventional radiation using fixed effect model

Postoperative accelerated RT did not result in a significant improvement of OS (n = 988, HR = 0.88, CI = 0.75–1.04, *p* = 0.148). The results are depicted in Fig. 6. Further analysis of the causes of death of the trial participants shows that accelerated radiation had not a significant effect in reducing cancer mortality (n = 988, HR = 0.83, CI = 0.68–1.02, *p* = 0.077) and nor on non-cancer related deaths (n = 988, HR = 0.98, CI = 0.74–1.30, *P* = 0.891) **(**Figs. 7 and 8**).**

In terms of side effects we summarized and categorized acute and late effects from the published trial results in Table 3. We found significantly more acute adverse events like mucositis and need for tube feeding in the patients treated with AF RT. The rate of late side effects did not significantly correlate with accelerated radiotherapy.

**Table 3:** Acute and late side effects

Trial	Ang et al. 2001 [9] *n* = 151			Awaad et al. 2002 [11] *n* = 70			Langendijk et al. 2015 [7] *n* = 148			Sanguineti et al. 2005 [8] *n* = 226			Suwinski et al. 2008 [10, 19] *n* = 275			CHARTWEL unpublished^a^ *n* = 114		
Acute Toxicity	CF	AF	p	CF	AF	p	CF	AF	p	CF	AF	p	CF	AF	p	CF	AF	p
Mucositis during RT ≥ grade II	n.r.	n.r.		67%	90%	0.04	n.r.	n.r.		74,7%	84,6%	0.007	n.r.	n.r.		n.r.	n.r.	
Mucositis during RT ≥ grade III	36%	62%	0.001	8%	16%		n.r.	n.r.		27%	53%		33.3%	60%	0.02	n.r.	n.r.	
Acute dysphagia ≥ grade III	n.r.	n.r.		10%	23%	0.007	n.r.	n.r.		6,3%	9,1%	n.c.	2%.	5%	n.c.	n.r.	n.r.	
Tube feeding	47%	51%	n.s.	n.r.	n.r.		n.r.	n.r.		8%	14%	0.13	*n* = 3	*n* = 5	n.c.	n.r.	n.r.	
Mean weight loss	n.r.	n.r.		n.r.	n.r.		n.r.	n.r.		0.9%	1.6%	0.3	3.3%	3.1%	n.c.	n.r.	n.r.	
Acute skin reaction, ≥ grade III	n.r.	n.r.		“Low in both groups ”		n.s.	n.r.	n.r.		35.1%	38.2%	n.c.	n.r.	n.r.		n.r.	n.r.	
Mucositis 6 weeks after RT	n.r.	n.r.		n.r.	n.r.		n.r.	n.r.		12%	9%	0.84	n.r.	n.r.		n.r.	n.r.	
Late toxicity:																		
Xerostomia grade II/III	n.r.	n.r.		39/13%	33/42%	0.17	n.r.	n.r.		*n* = 14	*n* = 16	n.c.	*n* = 14	*n* = 18	n.c.	n.r.	n.r.	
Lymphedema grade II/III	n.r.	n.r.		10%	16%	0.7	n.r.	n.r.		n.r.	n.r.		n.r.	n.r.		n.r.	n.r.	
Subcutaneous fibrosis or connective tissue grade II/III	n.r.	n.r.		13%	26%		n.r.	n.r.		*n* = 35	*n* = 36	n.c.	*n* = 13	*n* = 16	n.c.	n.r.	n.r.	
Dysphagia ≥ grade III	*n* = 13	*n* = 16	n.c.	n.r.	n.r.		n.r.	n.r.		*n* = 1	*n* = 3	n.c.	n.r.	n.r.		n.r.	n.r.	
Myelopathy, any grade	n.r.	n.r.		*n* = 0	*n* = 1	n.c.	n.r.	n.r.		*n* = 0	*n* = 1	n.c.	*n* = 0	*n* = 1	n.c.	n.r.	n.r.	
Any late side effects	No difference		n.s.	n.r.	n.r.		n.r.	n.r.		*n* = 49	*n* = 56	0.15	*n* = 51	*n* = 64	n.c.	n.r.	n.r.	
Any late side effects ≥ grade III	*n* = 25	*n* = 26	n.c.	n.r.	n.r.		n.r.	n.r.		*n* = 17	*n* = 24	n.c.	*n* = 8	*n* = 18	n.c.	n.r.	n.r.	
Any side effects							No difference		n.s.							n.r.	n.r.	

*CF* Conventional fractionation, *AF* Accelerated fractionation; values are reported as number of events or percent (%). *n.c.* Not calculated, *n.r.* Not reported, *n.s.* Not significant

^a^Data from the CHARTWEL trial were retrieved from [18]

We also performed a subgroup analysis comparing moderate accelerated radiation and very accelerated radiotherapy as defined by the MARCH Meta-analyses [14, 18]. We did not find a significant influence of these subgroups on any of the investigated endpoints (not reported in detail).

## Discussion

Despite advances in staging, surgical procedures, radiation techniques and systemic treatment options the clinical outcomes of patients with advanced HNSCC are still disappointing [20–26]. Radiobiological considerations as well as retrospective observational data rendered acceleration in head and neck radiation schedules as an attractive option to improve results [27, 28].

This meta-analysis addressed this question in the adjuvant setting using published effect sizes based on individual patient data with an average median follow-up of about 6 years. We found no significant effect of accelerated fractionated radiation compared to conventional fractionated radiation in the postoperative setting in any of the investigated endpoints.

The current standard of care for high risk HNSCC is concurrent adjuvant chemoradiation (CRT). This is based on the results of two large randomized trials using cisplatin concurrently with radiation [27, 28]. A pooled analysis of the two studies demonstrated a significant benefit in local control and overall survival especially in patients with close surgical margins or extracapsular lymphonodal extension [29].

Altered fractionation schedules as an alternative to systemic therapies to improve the therapeutic ratio in comparison to conventional RT have been intensively investigated. In the combined primary and postoperative setting Lacas et al. report that altered fractionation compared to conventional RT results in an improvement in almost all clinically important endpoints, including overall mortality (absolute difference at 5 years of 3.1%, CI 1.3–4.9) [18]. However alternate fractionation was associated with a significant increase in acute toxicity. In a subgroup analysis AF decreased local recurrence significantly. The mortality benefit though derived mainly from the trials using hyperfractionated instead of accelerated RT schedules.

In the sole primary setting of definitive radiotherapy Budach et al. previously published a meta-analysis assessing the effects of accelerated RT [30]. Over 10,000 patients were included in this analysis. They found a significant improvement in LRC with accelerated RT, but no significant benefit in OS. These results are in accordance to the MARCH meta-analysis by Bourhis and colleagues [14].

In the present meta-analysis we investigated whether the improvement in the primary setting for alternative fractionation translates into the postoperative setting. A subgroup analysis of timing of radiotherapy revealed no significant effect on OS or PFS in the updated MARCH meta-analysis [18]. However a complete analysis on postoperative accelerated RT had not been attempted. Our results showed that accelerated radiation fractionation does not significantly improve the studied endpoints. If any, the analyses of local control and cancer specific survival showed a minor trend for an effect of AF. These results are matching the established effects in the definitive radiation setting. Like in the updated MARCH meta-analysis AF has no effect on regional control despite a minor influence on local control. This is again in contrast to HF radiation schedules where its effects extend to both local and regional failures demonstrating a possible way to explain the superiority of HF to AF in survival endpoint.

Worsening of the acute toxicities is a well-established finding throughout the literature investigating alternative fractionation regimes [13, 18]. The analysis of the reported side-effects in Table 3 confirms a worsening of acute toxicities by AF. The acute mucositis rate was significantly higher and appeared earlier during treatment in the accelerated fractionated RT group. Similarly to other trials and meta-analyses we also did not find an increase in late side effects. Therefore prior surgery does not appear to have a noticeable influence on the pattern of adverse events.

This meta-analysis has some strengths as well as limitations to address. The average median trial follow-up of about 6 years provides robust long-term oncological results. The use of pooled trial results of individual patient data allows for a more sophisticated estimation method compared to extracting hazard ratios from published survival curves. Yet the clinical applicability is limited by the relatively small number of analyzable patients (*n* = 988). Certain analyses might be underpowered to show a benefit of AF in postoperative radiation. Furthermore we were unable to perform any subgroup analysis. Especially as p16-status, a surrogate marker for human papilloma virus (HPV) infection, is an important prognostic marker in HNSCC, studying the effect on fractionation with respect to HPV status would be interesting. Moreover all included trials used conventional radiation techniques which are currently non-standard with the introduction of volumetric arc therapy and image-guided radiation therapy. Another limitation is that the median follow-up time is unequal between the included trials which might lead to heterogeneity in the outcome of the clinical endpoints.

Concurrent chemoradiation or hyperfractionated radiation therapy is both an accepted standard in the definitive treatment of HNSCC and was indirectly shown to be equally effective [31]. Adding chemotherapy to HF RT schedules could provide additional benefits [32, 33]. However this has not been demonstrated for AF [12]. At least in the primary setting, HF, and not AF, should therefore be used as the preferred alternate fractionation schedule. However the Radiation Therapy Oncology Group (RTOG) has implemented a moderately accelerated RT schedule in their comparison of cisplatin vs. cetuximab (RTOG 1016).

In the adjuvant situation the comparison of AF and HF to CRT has not been successfully reported. There is equally a lack of data for adjuvant HF RT compared to standard RT schedules which leaves CRT, CF RT and AF RT as studied options. The clinical scenario where one might consider an accelerated postoperative radiation schedule is difficult to identify. As chemotherapy is very likely superior to adjuvant accelerated radiation only patients who are unable to tolerate concurrent CRT are possible candidates. Further they would need to be at very high risk of local failure and additionally be able to comply with the accompanied aggravated acute toxicities. Our study was not able to identify any potential subgroup that could derive a profound profit from AF.

Future studies using modern RT techniques and simultaneous integrated boost (SIB) might improve the risk benefit ratio in this setting. Intensity modulated radiotherapy was already shown to improve acute and late toxicities compared to conventional RT [34, 35]. Likewise SIB might add a reduction in the incidence of late adverse events compared to sequential boost techniques [36].

## Conclusion

Concurrent chemoradiotherapy (conventionally-fractionated) is the standard-of-care as adjuvant intervention for resected and high-risk HNSCC. Postoperative accelerated radiotherapy does not result in a significant improvement of overall survival in high-risk patients. Clinical decision of adjuvant accelerated radiotherapy must be made on case to case basis weighing overall risks and benefits. Future studies with the use of modern radiation techniques might change the risk-benefit ratio in the treatment of squamous cell carcinoma of the head and neck.

## Abbreviations

AF: Accelerated fractionation
CF: Conventional fractionation
CI: 95% confidence interval
CL: Confindence limits
CRT: Chemoradiation
ECE: Extracapsular extension
HF: Hyperfractionated
HNSCC: Squamous cell carcinomas of the head and neck
HR: Hazard ratio
IGRT: Image-guided radiation therapy
IMRT: Intensity-modulated radiation therapy
LRC: Locoregional control
n.c.: Not calculated
n.r.: Not reported
n.s.: Not significant
OS: Overall survival rate
QoL: Quality of life
RT: Radiotherapy
